# Analysis of inflammatory markers in apparently healthy automobile vehicle drivers in response to exposure to traffic pollution fumes

**DOI:** 10.12669/pjms.36.4.2025

**Published:** 2020

**Authors:** Hina Riaz, Binafsha Manzoor Syed, Zulfiqar Laghari, Suleman Pirzada

**Affiliations:** 1 Dr. Hina Riaz, MBBS, Lecturer, Department of Physiology, Liaquat University of Medical & Health Sciences (LUMHS), Jamshoro, Pakistan; 2 Dr. Binafsha Manzoor Syed, MBBS, PhD, Director Medical Research Centre, Director Clinical Research Division, Director ORIC, Liaquat University of Medical & Health Sciences (LUMHS), Jamshoro, Pakistan; 3 Prof. Dr. Zulfiqar Laghari, PhD, Chairperson, Department of Physiology, University of Sindh, Jamshoro, Pakistan; 4 Dr. Suleman Peerzada, MBBS, PhD, Assistant Professor, Department of Molecular Biology and Genetics, Liaquat University of Medical & Health Sciences (LUMHS), Jamshoro, Pakistan

**Keywords:** Traffic pollution, Healthy drivers, Markers of inflammation

## Abstract

**Objective:**

This study aimed to evaluate pattern of markers of inflammation in apparently healthy drivers who exposed to traffic fumes.

**Methods:**

This cross-sectional study was conducted from June 2016 to January 2017 at Liaquat University of Medical & Health Sciences (LUMHS), Jamshoro. It looked into the effects of traffic pollutants on markers of inflammation including CRP, Leukocytes count, IL-6, TNF-α, TNF-β of healthy human volunteers. Eighty-seven, apparently healthy, non-smoking automobile vehicle drivers, having daily contact of traffic exhaust for at least six hours, aged between 18-40 years recruited for this study. Levels of traffic-generated pollutants P.M_2.5_, P.M_10,_ NO_x_ were recorded in different areas of Hyderabad City.

**Results:**

P.M_2.5_ found to be positively correlated with markers of inflammation including IL-6 (*r_s_* = 0.99), TNF-α (*r_s_* = 0.41), CRP mg/dl (*r_s_* = 0.99) , neutrophils (*r_s_* = 0.29), lymphocytes (*r_s_* = 0.31), eosinophils (*r_s_* = 0.20), monocytes (*r_s_* = 0.42) and basophils (*r_s_* = 0.16). Positive correlation present among IL-6 (*r_s_* = 0.21), TNF-α (*r_s_* = 0.49) and CRP mg/dl (*r_s_* = 0.22) % (*r_s_* = -0.31), Leukocytes (*r_s_* = 0.14) neutrophils (*r_s_* = 0.31), lymphocytes (*r_s_* = 0.21), monocytes (*r_s_* = 0.50), basophils (*r_s_* = 0.17) with P.M_10_. NO_x_ showed positive correlation with IL-6 (*r_s_* = 0.22), TNF-α (*r_s_* = 0.48), CRP (*r_s_* = 0.22), neutrophils (*r_s_* = 0.31), lymphocytes (*r_s_* = 0.13), basophils (*r_s_* = 0.17) and monocytes (*r_s_* = 0.48).

**Conclusion:**

Findings of our study suggest that almost all markers of inflammation are positively correlated with traffic pollutants and this condition might raise the risk of systemic diseases.

## INTRODUCTION

Pakistan has been ranked among highly polluted countries of the world causing drastic effects on health, posing economic burden on the country.^1^,^2^ World air quality index 2019, reported that Lahore is the second and Karachi is the seventh most polluted cities of the world, recorded particulate matter (PM) concentration in Lahore and Karachi were 188 ug/m^3^, 153 ug/m^3^ respectively. Approximately seven million early deaths have been reported around due to air pollution. Smog is the worst form of pollution comprised of fog, smoke of burning crops and traffic generated pollutants; every year Lahore is submerged with smog with first winter spell.^2^,^3^

Exposure to traffic pollutants such as PM_2.5_, PM_10_ are associated with rise in mortality rate due to lung cancer and cardiovascular diseases.^4^ Study reported that prolong exposure to PM_10_, nitrogen dioxide (NO_2),_ sulphur dioxide (SO_2_), carbon monoxide (CO), is related with dyslipidemia, cardiac diseases (ischemic heart diseases, myocardial infarction) and diabetes mellitus.^5^ PM is one of the causative agents of neurological diseases by potentiating the oxidative damage in neural vasculature.^6^ Maternal Contact to PM_2.5,_ CO, SO_2_ and oxides of nitrogen NO_x_ is associated with preterm deliveries and low birth weight of infants and congenital abnormalities.^7^,^8^ Moreover prolong exposure to PM_10_ and NO_2_ is a causative factor of deaths in middle aged women due to cardiopulmonary diseases.^9^ It is reported by study that exposure of diesel exhaust potentiate the inflammatory process in human body which results in chronic diseases like asthma, cardiopulmonary disorders and cancers specially in susceptible population (children and elderly).^10^,^11^ Exposure of diesel exhaust fumes in infancy causes changes in gene expression of TNF, IL-10 and IL-13.^12^

As the traffic fume pollution is a global health concern, drastically affecting developing countries including Pakistan. however limited data is available till date to look into the pattern of markers of inflammation in apparently healthy population. Thus, this study was designed to evaluate the pattern of inflammatory markers in apparently healthy drivers who were exposed to traffic fumes. To the best of our knowledge, this is preliminary study in Pakistan that is addressing the effects of traffic exhaust fumes on human health.

## METHODS

This cross-sectional study conducted from June 2016 to January 2017 at Liaquat University of Medical & Health Sciences (LUMHS), Jamshoro. It looked into the effects of traffic pollutants on markers of inflammation of apparently healthy automobile vehicle drivers. Markers of inflammation including: C-reactive protein (CRP), Leukocytes count, interleukin-6 (IL-6), tumor necrotic factor-α (TNF-α) and tumor necrotic factor-β (TNF-β), total leukocyte and differential leukocytes count (neutrophils, lymphocytes, eosinophils, monocytes and basophils). For this study eighty-seven, apparently healthy, non-smoking automobile vehicle drivers, having daily contact of traffic exhaust for at least six hours, aged between 18-40 years were recruited. Non-smoking, apparently healthy, not suffering from any systemic disease or autoimmune diseases volunteers were included in this study. While volunteers suffering from any systemic disorders, autoimmune diseases, overweight person (BMI ≥30 kg/m^2^) were not considered.

### Methods of Inflammatory markers analysis

3 ml of whole blood was drawn from all the volunteers for analysis of inflammatory markers. CRP analyzed by “C-reactive protein Hitachi 902 turbidemetry “and total leukocytes count by “Automated Analyazer” (sysmex). While IL-6, TNF-α and TNF-β were analyzed by “human instant ELISA (enzyme linked immunosorbent assay) (KOMA Biotech) ^®^ kits for analysis of markers. The standard laboratory method was followed, briefly 200 ul of washing solution added to each well. Wells were aspired and excessive liquid removed 100 ul of standard (sample) then incubated at room temperature for two hours. Well aspirated and washed. 100 ul of diluted detection antibody (0.4 ug/ml for TNF-α, 0.1ug/ml for TNF-β and for IL-6 0.25ug/ml) to each well covered with the plate sealer then incubated for two hours then diluted Color Development Enzyme (1:20 dilute) each well was added. Incubated at room temperature until the appropriate color development at least for 17-27 minutes, plate read at 450 nm wavelength.

### Exposure analysis

Measurement of pollutant (P.M_2.5,_ P.M_10_, and NOx) carried out at seventeen busy areas of Hyderabad (Sindh), where traffic flow is usually heavy. P.M Meter (Model Aerocet: 531) used for the analysis of PM_2.5_ and P.M_10_ and NO_x_ Meter (Model: AC32M) for NO_x_ Level of pollutants compared with National Environmental Air Quality Standards (NEAQS). Concentration of each pollutant recorded at each location on three consecutive days of six months. Short duration exposure cut off value was five years (≤ 5), while uppermost limit was twenty-five years (≥25).

### Statistical analysis

Data analyzed by using IBM statistical program for social sciences (SPSS), (IBM Corp. Released 2012. IBM SPSS Statistics for Windows, Version 23.0. Armonk, NY: IBM Corp. version) Spearman rank correlation used to determine the correlation of traffic pollutant with markers of inflammation. The results of all analyses evaluated for statistical significance using p-value < 0.05 and the 95 % confidence intervals (CI).

### Ethical consideration

The project was approved by the Ethical Committee of Liaquat University of Medical and Health Sciences (Ref No. LUMHS/REC/94, dated Oct. 8, 2013) under the title: Detrimental Effects of Air Pollution on systemic health, Lung Volumes and Capacities of Young, Healthy Pakistani Volunteers.

## RESULTS

During study period, 87 apparently healthy subjects included in the study. Mean age of subjects was 31(±7.3) years. While mean height and mean weight were, 174cm (±4.8) and, 75 kg (±6.3) respectively. Mean Body mass index (BMI) was, 24.3 (±2.4). Mean concentration of PM_2.5_ (39.38), PM_10_ (254.18) and NO_x_ (37.26) recorded at different busy locations of city where traffic flow is quite high, compared with standard NEQS, as shown in Table-I mean levels of markers of inflammation are mentioned in Table-II.

**Table-I:** Mean concentration of pollutants of busy areas of Hyderabad City.

Pollutants	P.M2.5 ug/m^3^	P.M10 ug/m^3^	NOxug/m^3^
NEAQS Levels	35	150	80
Jamshoro level crossing	38.16	240.7	32.56
Qasim Intersection	37.8	363.4	27.8
Qasimabad	40.16	263.6	40.5
Civil Hospital	43.4	234.1	35.6
Hyder intersection	42.3	257.3	28.5
Chandni shopping market	44.2	233.5	41.23
Giddu intersection	38.96	299.50	40.23

**Table-II:** Mean levels of markers of inflammation.

Markers of inflammation	Mean
TNF-α pg/ml	7.7
IL-6 pg/ml	60
TNF-β pg/ml	29
CRP mg/dl	0.66
Leukocytes	9.1
Neutrophils%	54
Eosinophils %	5.0
Basophils%	2.1
Lymphocytes%	28
Monocytes %	6.1

P.M_2.5._ found to be positively correlated with following markers of inflammation; IL-6 (*r_s_* = 0.99, p = 0.001), TNF-α (*r_s_* = 0.41, p = 0.001), CRP mg/dl (*r_s_* = 0.99, p = 0.001) , neutrophils (*r_s_* = 0.29, p-value = 0.006), lymphocytes (*r_s_* = 0.31, p-value = 0.003), eosinophils (*r_s_* = 0.20, p-value = 0.06), monocytes (*r_s_* = 0.42 p-value = 0.001) and basophils (*r_s_* = 0.16, p-value = 0.14). While, there were no correlations present with total Leukocytes count (*r_s_* = 0.03, p-value = 0.73), and TNF-β (*r_s_* = 0.47 p-value = 0.66) as shown in, Table-III.

**Table-III:** Spearmen Correlation in markers of inflammation and P.M_2.5_

Markers of Inflammation	P.M_2.5_ug/m^3^	

	r_s_	p –value
TNF-αpg/ml	0.41	0.001
IL-6pg/ml	0.99	0.001
TNF-βpg/ml	0.47	0.66
CRP mg/dl	0.99	0.001
Leukocytes	0.03	0.73
Neutrophils	0.29	0.006
Eosinophils	0.20	0.006
Basophils	0.10	0.14
Lymphocytes	0.31	0.003
Monocytes	0.42	0.001

There was a positive correlation present among IL-6 (*r_s_* = 0.21, p = 0.04), TNF-α (*r_s_* = 0.49, p = 0.001), CRP mg/dl (*r_s_* = 0.22, p = 0.03), Leukocytes (*r_s_* = 0.14, p-value = 0.17) neutrophils % (*r_s_* = 0.31, p-value = 0.003), lymphocytes % (*r_s_* = 0.21, p-value = 0.042), monocytes % (*r_s_* = 0.50, p-value = 0.001), basophils % (*r_s_* = 0.17, p-value = 0.11) with P.M_10_. Whereas there was no correlation present with TNF-β (*r_s_* = 0.017, p = 0.66) and eosinophils % (*r_s_* = 0.09, p-value = 0.37), as shown in, Table-IV.

**Table-IV:** Spearmen Correlation among markers of inflammation and P.M_10_

Markers of Inflammation	P.M_10_ug/m^3^	

	r_s_	p –value
TNF-αpg/ml	0.49	0.001
IL-6pg/ml	0.21	0.04
TNF-βpg/ml	0.01	0.66
CRP mg/dl	0.22	0.03
Leukocytes	0.14	0.17
Neutrophils%	0.31	0.003
Eosinophils %	0.06	0.37
Basophils%	0.17	0.11
Lymphocytes%	0.21	0.042
Monocytes %	0.50	0.001

Markers of inflammation showed positive correlation with NO_x_ like; IL-6 (*r_s_* = 0.22,p = 0.03), TNF-α (*r_s_* = 0.48, p = 0.001), CRP (*r_s_* = 0.22, p = 0.03), leukocytes (*r_s=_ 0.14, p = 0.18)* neutrophils (*r_s_* = 0.31, p = 0.003), lymphocytes (*r_s_* = 0.13, p = 0.22), eiosinophils (*r_s =_ 0.10 p = 0.34)*, basophils (*r_s_* = 0.17, p = 0.10), lymphocytes and monocytes (*r_s_* = 0.48, p = 0.001). While no correlation was found with TNF-β as shown in, Table-V.

**Table-V:** Spearmen rank Correlation among markers of inflammation and NOx.

Markers of Inflammation	NOxug/m^3^	

	r_s_	p –value
TNF-αpg/ml	0.48	0.001
IL-6pg/ml	0.22	0.03
TNF-βpg/ml	0.02	0.82
CRP mg/dl	0.22	0.03
Leukocytes	0.14	0.18
Neutrophils%	0.31	0.003
Eosinophils %	0.10	0.34
Basophils%	0.17	0.10
Lymphocytes	0.13	0.22
Monocytes	0.48	0.001

## DISCUSSION

According to findings of our study, most of markers of inflammation showed positive correlation with pollutants, Our study results have elaborated the Th1/Th2 derived pro inflammatory cytokines (TNF-α, TNF-β and IL-6), since the study population was healthy subjects increase in serum concentration at subclinical level is a frightening condition given that constant rise of inflammatory markers can lead to systemic diseases including autoimmune disease and even cancer. PM is highly hazardous for human health it is a combination of sulfate, sodium chloride, ammonia, mineral dust and black carbon. Due to its micro size it get lodge deep into lungs, along with NO_x_ it causes irritation of respiratory mucosa and initiate local inflammation (TNF-α, IL-6), diffusion of markers of inflammation transported into circulation and mediate systemic inflammatory cascade. Effects of traffic pollutants on respiration already discussed in a separate chapter of thesis.

In our study CRP showed positive significant correlation with pollutants PM_2.5_ and PM_10_, these findings are consistent with the results of Pilz (2018) study, in which PM_2.5_, PM_10_, NO_2_, and NO_x_ showed positive association with CRP on long term exposure.^13^ Similar results by Lee (2018), reported that short term exposure of pollutant like SO_2_, NO_2_ and CO causes increase in fibrinogen level in non-smokers while, long term exposure causes rise in fibrinogen white blood cell (WBC) concentration.^14^ Study also revealed that short term exposure NO_2_ of non-smoking, healthy subject showed weak association with of markers of inflammation (IL-6,CRP, TNF-α) while strong positive association found on long term exposure among non-smokers and physically healthy subjects.^15^ In our study monocytes showed significant positive correlation with PM, NOx and CO while increase in monocytes concentration at subclinical level after exposure to traffic related NO_2_ and PM as reported by another study.^16^ Another study reported that both coarse PM_10_ and fine PM_2.5_ enhanced monocytes antigen presenting capacity.^17^ PM_10_ exposure to monocytes triggers increase intracellular calcium^18^. Exposure of monocytes to nano particle of black carbon causes release of pro inflammatory cytokines (TNF-α, IL-6, IL-8) and increase in phagocytic capability of monocytes.^19^

Furthermore, diabetogenic biomarkers (adiponectin, interleukin-1 and CRP) found to be elevated in healthy non diabetic persons on short and long duration exposure of PM_2.5_, PM_10_ and NO_2_.^20^ In addition, one more study documented that PM_2.5_ exposure causes increase in serum concentration of CRP and neutrophils in non-smoking healthy subjects 21. Likewise a Nepali study revealed positive association of CRP with PM_2.5_ while, negative association reported with TNF-α and IL-6.^22^ On the contrary a Belgian study reported no effects on total leukocytes concentration of healthy subject on exposure of traffic fumes and traffic related benzene causes decrease in WBC count, lymphocytes, eosinophils and platelets.^23^,^24^ Yet another study reported that when healthy female subjects exposed to pollutants for short duration CRP and leukocytes did not show positive association.^25^ A German study also stated prolong residential exposure of pollutant (PM_2.5_), showed weak association with CRP and no any association on short duration exposure in non-smoking volunteers,^26^ in the same way a Californian study reported association of TNF-α, IL-6 and CRP with NO_x_ and CO.^27^ Association of TNF-α with NO_x_ documented by another study.^28^ In our CRP, IL-6 and TNF-α showed positive correlation with PM_10_, on the other hand Tsai (2019) conducted study in Switzerland exposed the general population to PM_10_ at low concentration; reported positive association of similar marker of inflammation while no significant association with CRP.^29^

### Limitations of the Study

It incuded unavailability of continous air quality data, small smaple size and cross-sectional study design. However prospective nature of the study is a major strength besides having assessed environmental pollution at different areas of the study in order to confirm the exposure of the pollutant.

## CONCLUSION

Findings of our study suggest that almost all markers of inflammation are positively correlated with traffic generated pollutants this condition might raises the risk of systemic diseases and causing deterioration health status of apparently of healthy subjects.

### Recommendation

Pakistan has been ranked top most polluted countries of the world; at present country is facing distressing air quality index. Quite a few studies had reported air quality status of major cities of Pakistan but still lacking consistent data of country. There is critical need of such research projects on broad scale and large sample size. Joint projects with collaboration of environmental protection department should be conducted on urgent basis.

### Author`s Contribution

**BMS, ZL and SP** are supervisors of **HR**.

**HR** conceived, designed, data collection and did statistical analysis & editing of manuscript, will be responsible and accountable for the accuracy or integrity of the work.

**BMS** supervised project, did review and final approval, editing of manuscript.

**ZL** supervised project, did review and final approval.

**SP** did review of manuscript.
